# ASO Author Reflections: Reevaluating Treatment Sequence in Metaplastic Breast Cancer

**DOI:** 10.1245/s10434-025-18316-3

**Published:** 2025-09-09

**Authors:** Margo Nelis, Anna Chichura, Zahraa Al-Hilli

**Affiliations:** 1 Obstetrics and Gynecology Institute, Cleveland Clinic, Cleveland, OH USA; 2 Division of Surgical Oncology, Department of Surgery, University of Illinois at Chicago, Chicago, IL USA; 3 Division of Gynecologic Oncology, Department of Obstetrics and Gynecology, University of Illinois at Chicago, Chicago, IL USA; 4 Breast Center, Integrated Surgical Institute, Cleveland Clinic, Cleveland, OH USA

## Past

Metaplastic breast cancer (MpBC) is a rare and aggressive subtype of breast cancer that frequently exhibits triple-negative receptor status and carries a worse prognosis than other morphologic subtypes of triple-negative breast cancer (TNBC).^1^ The standard of care for locally advanced TNBC is neoadjuvant chemotherapy (NAC).^2^ While the sequence of chemotherapy and surgery is not believed to impact overall survival (OS), NAC offers potential advantages, including the opportunity to deescalate surgery, obtain prognostic information, and individualize adjuvant systemic therapy.^3^

## Present

In a large National Cancer Database (NCDB)-based analysis of more than 5000 MpBC cases, our study demonstrated that patients who underwent upfront surgery followed by adjuvant chemotherapy had significantly improved 5-year overall survival compared with those receiving NAC (HR 0.556, 95% CI 0.464–0.667; *p* < 0.0001).^4^ The pathologic complete response rate (PCR) was low (4.9%), and NAC did not improve the rate of breast conservation (33.25% upfront surgery versus 49.53% NAC, *p* < 0.0001). Prior to this study, the impact of treatment sequencing in MpBC had not been well characterized, making these findings particularly impactful. This analysis reinforces the limited effectiveness of NAC in MpBC and supports surgical resection as the preferred initial approach in most cases.

## Future

While upfront surgery for MpBC has been associated with superior outcomes—particularly when compared with neoadjuvant chemotherapy—the potential of neoadjuvant chemoimmunotherapy or other novel neoadjuvant strategies to improve PCR rates remain uncertain. The addition of immunotherapy to standard chemotherapy has been shown to enhance treatment efficacy in other subtypes of TNBC, but the applicability of these findings to MpBC has yet to be determined.^5^ Future prospective studies are needed to evaluate the response of metaplastic TNBC to neoadjuvant chemoimmunotherapy, advance the development of molecularly targeted therapeutics, and define precision treatment strategies tailored to the distinct biologic and molecular characteristics of the various MpBC subtypes.
